# Myocardial strain analysis as a non-invasive screening test in the diagnosis of stable coronary artery disease

**DOI:** 10.1186/s43044-021-00173-6

**Published:** 2021-05-25

**Authors:** Nehzat Akiash, Mohammad Mohammadi, Hoda Mombeini, Akbar Nikpajouh

**Affiliations:** 1 Atherosclerosis Research Center, Ahvaz Jundishapur University of Medical Sciences, Ahvaz, Iran; 2 Rajaie Cardiovascular Medical and Research Center, Iran University of Medical Sciences, Tehran, Iran

**Keywords:** CAD, Strain analysis, 2D-STE, non-invasive tests, Echocardiography

## Abstract

**Background:**

Coronary artery disease (CAD) is one of the most prevalent diseases around the world; however, finding the best noninvasive, low-cost, and more easily accessible test for its screening has been a challenge for several years. Eighty-nine patients suspected of stable CAD underwent 2D-speckle-tracking echocardiography (2DSTE) at resting position and offline longitudinal myocardial strain analysis, followed by coronary angiography. The correlation of the global longitudinal strain (GLS) and territorial longitudinal strain (TLS) with significant CAD (70% and more stenosis in at least one coronary artery) was then evaluated.

**Results:**

The statistical analysis showed a significant correlation between low GLS and significant CAD (P=0.0001). The results also showed a significant correlation between low TLS and significant CAD in the left and right coronary artery territories. The optimal cut-off point of GLS for the detection of significant CAD was −19.25, with a sensitivity of 76.5% and specificity of 76.6%.

**Conclusion:**

This study confirmed the usefulness of 2DSTE myocardial strain analysis in diagnosis of CAD for detecting the affected coronary arteries using GLS and SLS.

## Background

Coronary artery disease (CAD) is one of the most prevalent disorder that is further growing as the population grows old. Chest pain accounts for six million emergency room visits in the USA every year and leading to ten million stress tests and one million coronary angiographies [1, 2].

Finding the best noninvasive screening test for CAD is still a matter of debate [3]. Exercise tests are the first-line screening for most patients, but lower sensitivity and specificity sometimes limited them in CAD diagnosis even combined with myocardial perfusion imaging or echocardiogram. Furthermore, these tests are not practical for patients with mobility issues [4, 5]. On the other hand, myocardial perfusion scans and CT angiography are expensive and pose the risk of radiation exposure [5].

Myocardial strain analysis with 2D-speckle-tracking echocardiography (2DSTE), which is performed at resting position, offers valuable information about myocardial fiber changes in different types of myocardial diseases [6–8]. Speckle tracking consists of analyzing the motion of the speckles within selected segments of the myocardium, combined with an analysis of myocardial segment changes in strain is a state-of-the-art method for the diagnosis of subclinical diseases. Strain changes occur in three dimensions: longitudinal, radial, and circumferential. True strain as myocardial lengthening and shortening is a complex three-dimensional deformation, also encompassing rotation. What is measured with strain by echo is a substitute for this complex deformation [8, 9]. Despite Doppler tissue imaging (DTI), 2DSTE is not influenced by noises or adjacent segment tethering and is also not angle-dependent [10–13]. Subendocardial myocardial fibers are the most sensitive areas to ischemia, so that measuring the changes in their length and figures can provide the most sensitive indicator of ischemia arising from CAD [14, 15] [1, 16]. This study evaluates the sensitivity and specificity of this new modality for screening CAD in patients suspected of having stable angina pectoris.

## Methods

### Study design

The exclusion criteria were considered as following age below 18 years, a left ventricular ejection fraction <50% based on the 2D echocardiography, a prior history of CAD, myocardial infarction, percutaneous coronary intervention (PCI) or coronary artery bypass graft, acute coronary syndrome (ACS), congestive heart failure, any wall motion abnormality, more than mild valvular heart disease, ventricular conduction disturbances, pathological Q waves in resting ECG, atrial fibrillation, poor image quality for assessing all the segments by speckle tracking, and having refused coronary angiography.

### Standard echocardiography and speckle-tracking strain analysis

Upon admission, the patients’ complete medical history was taken, and they underwent a physical examination and 2D echocardiography for speckle-tracking evaluation and conventional transthoracic echocardiography at resting position using Vivid E9 ultrasound (USA, GE Ultrasound). Echocardiographic studies were performed and analyzed by a trained fellow of advanced echocardiography who was blinded to the coronary angiography results.

**Fig. 1:**
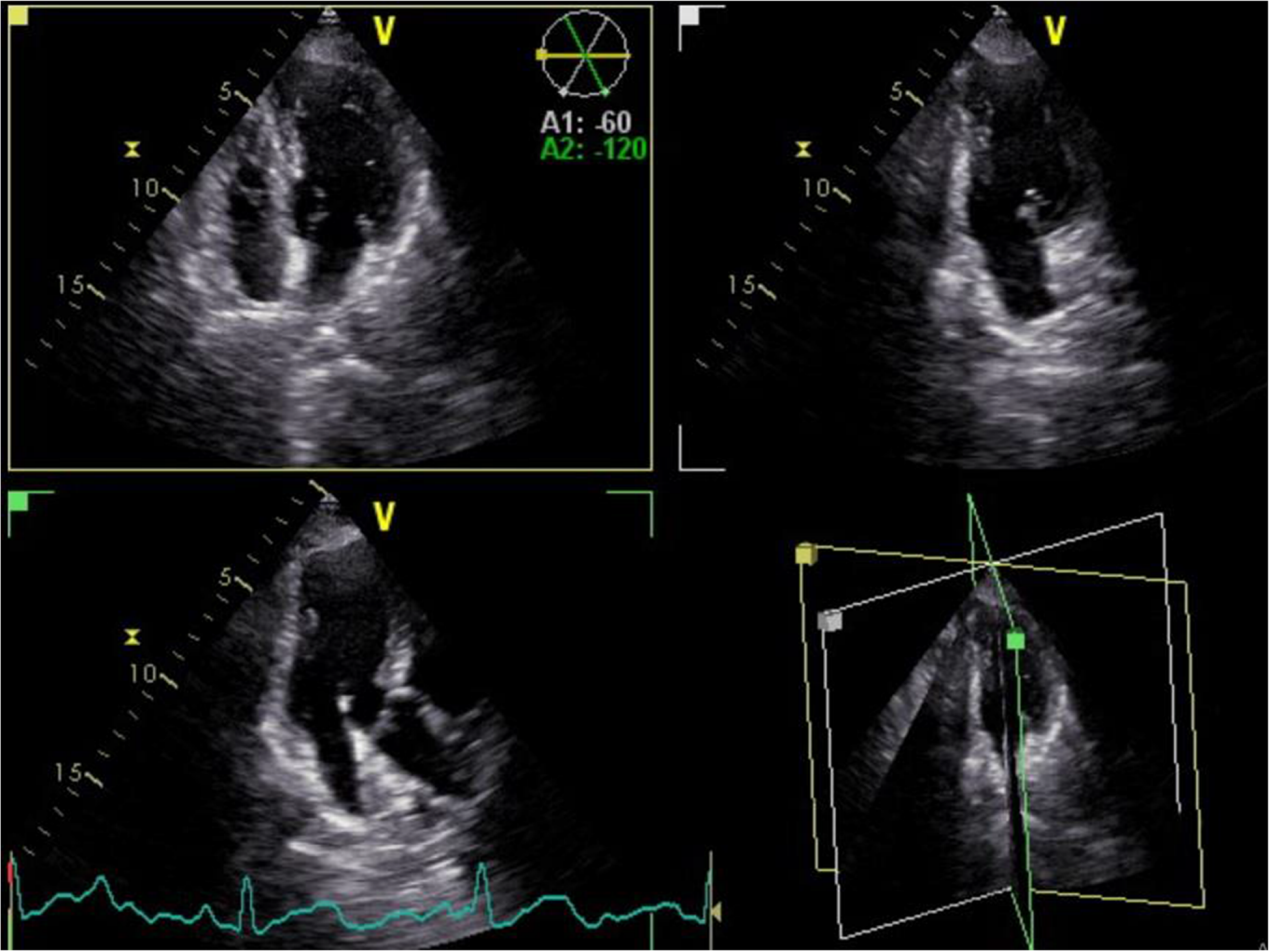
Three standard views, two-, three-, and apical four-chamber, for evaluation of longitudinal strain

For longitudinal strain evaluation, three standards (apical two-, three-, and four-chamber) views were recorded from three consecutive beats (Fig. 1) and the offline longitudinal strain analysis was performed using the Automated Function Imaging (AFI) model (GE Ultrasound) by two echocardiologists separately who were expert in strain analysis.

**Fig. 2:**
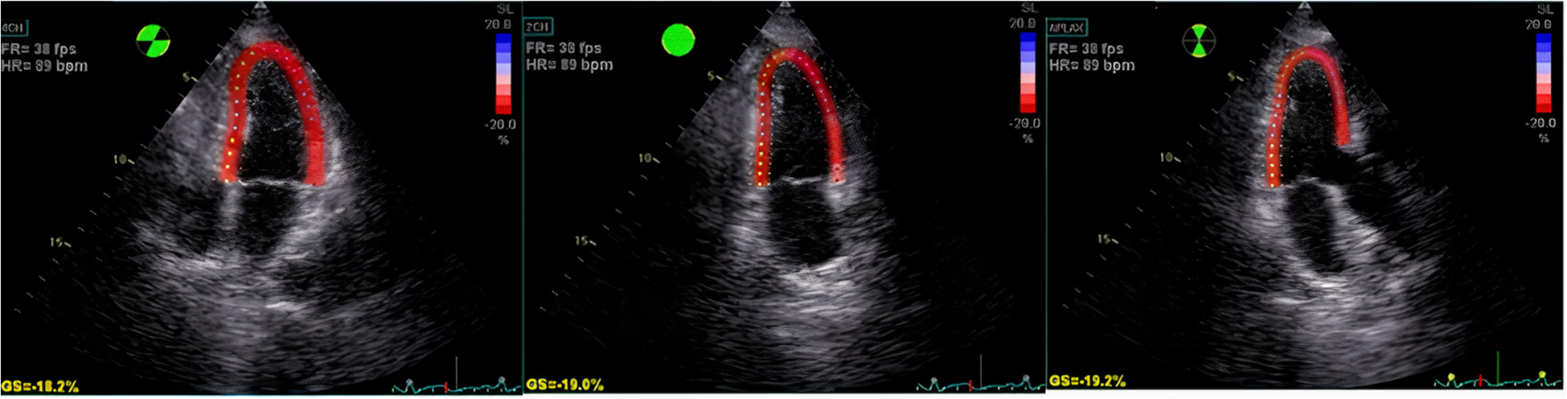
LV contour was traced in each view for evaluation of longitudinal strain score to each myocardial segment

In this method, the LV contour was traced in each view, and after some manual endocardial adjustment, the software automatically gave a longitudinal strain score to each myocardial segment (Fig. 2).

**Fig. 3:**
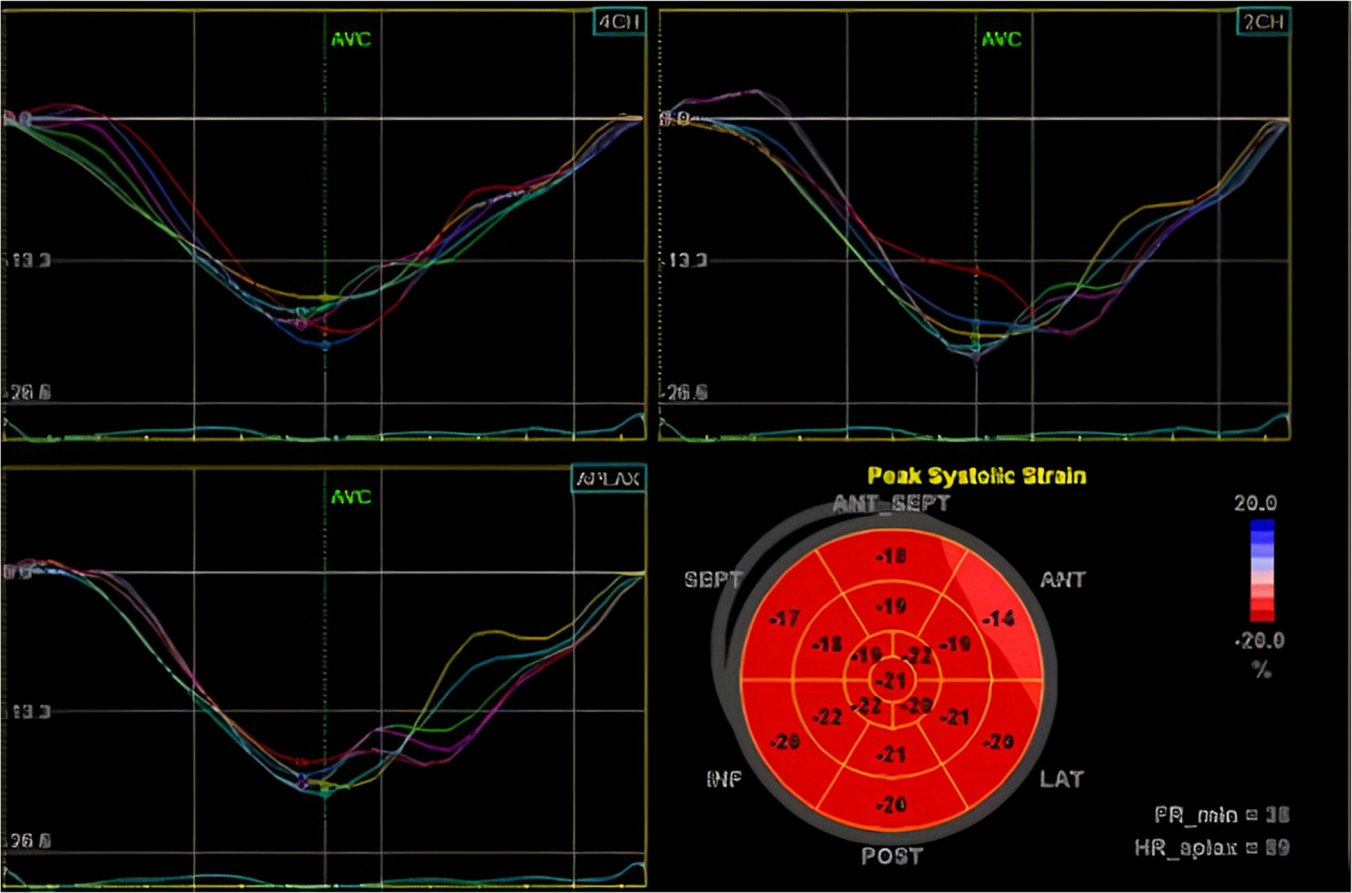
Bull’s eye map of each segment with strain curves

**Fig. 4:**
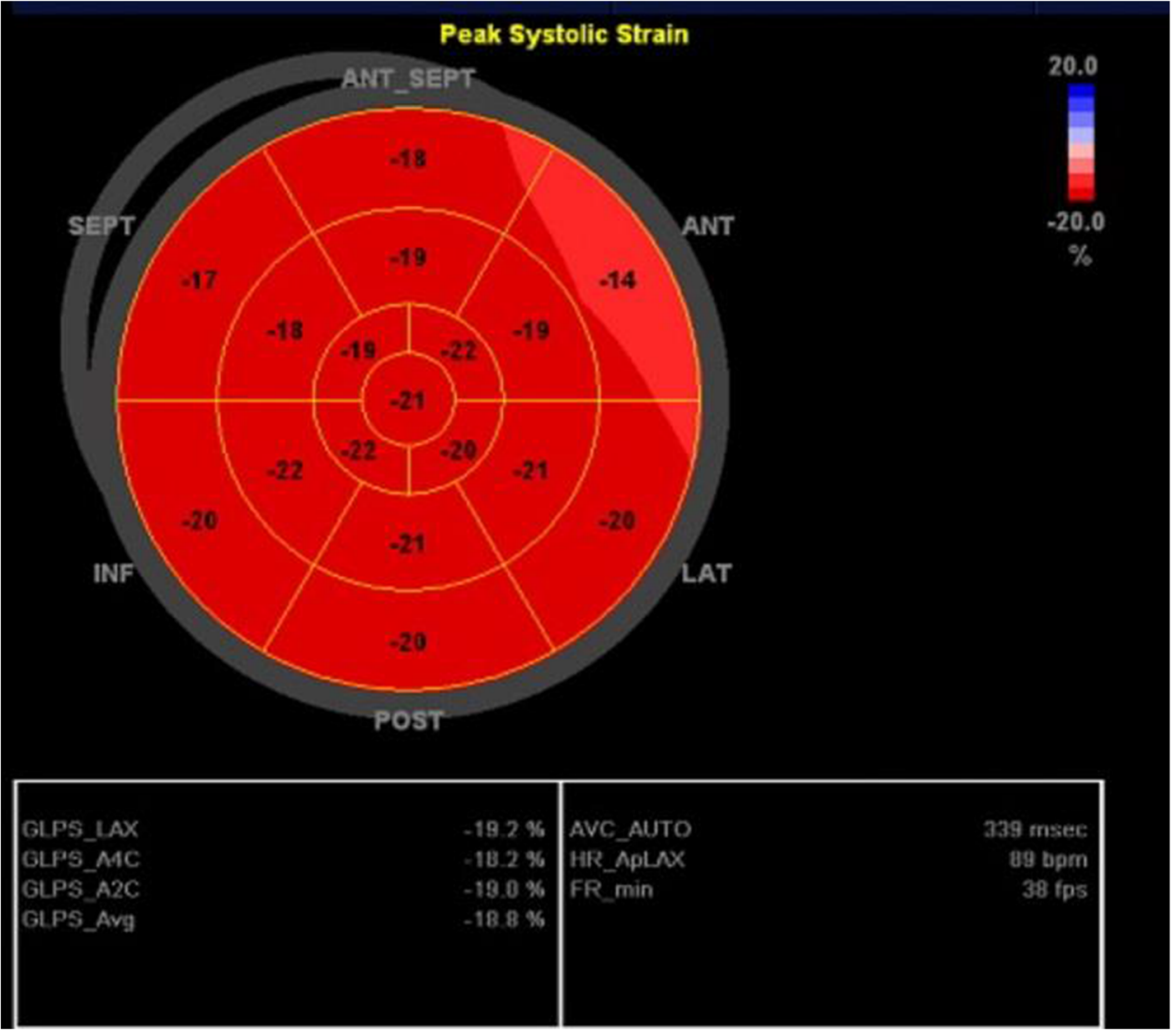
Bull’s eye map of each segment with global longitudinal strain (GLS) of 2, 3, and 4 chamber views and average GLS

Finally, peak systolic longitudinal strain values were recorded for each segment in the form of 17-segment bull’s eye that was labeled as segmental longitudinal strain (SLS). Global longitudinal strain (GLS) was automatically calculated as the mean value of three apical projections (Figs. 3 and 4).

Territorial longitudinal strain (TLS) was calculated for three major coronary arteries (LAD, LCX, and RCA) by the mean value of SLS in segments perfused by each coronary artery. We applied 17-myocardial segment popular pattern which includes 7 segments related to LAD (basal anterior, basal anteroseptal, mid anterior, mid anteroseptal, anthropical, apical septal, apex cap), six segments related to LCX (basal lateral, basal posterior, mid posterior, mid lateral, apicolateral), and six RCA-related segments (basal septal, inferobasal, mid inferior, mid septal, inferoapical).

### Coronary angiography

All the patients underwent coronary artery angiography within 1 week of the echocardiography by an interventional cardiologist, who was blinded to the echocardiography reports. Angiograms were performed via the femoral or radial artery approach, and at least two projections were made for each coronary vessel. Seventy percent and more stenosis in at least one coronary artery, including the left anterior descending (LAD) and its large branches (i.e., diagonals), the left circumflex coronary artery and its large branches (i.e., obtuse marginal [OM] branch), and the right coronary artery (RCA), and 50% and more stenosis in the left main (LM) coronary artery were taken as significant CAD. Stenosis between 50 and 70% in the coronary arteries (except LM) was taken as moderate CAD, and stenosis less than 50% was taken as mild CAD.

### Statistical analysis

The results of the 2DSTE and coronary angiograms were statically analyzed in SPSS software (version 25). The T-test was used to evaluate the correlation between the strain scores and significance of CAD. A ROC curve analysis was performed to predict the cut-off point of GLS for the best sensitivity and specificity for predicting significant CAD.

## Results

Ninety-four patients were finally enrolled in this study. Five of them were excluded due to unsatisfactory image quality for strain analysis. The mean age of 89 patients was 57.3 years, with a range of 34 to 88 years. Fifty-six of them were male (62.9%), and 33 (37.1%) were female. Table 1 represents the detailed demographic data of the patients.

**Table 1:** Patients’ characteristics

Patients’ characteristics	Result
Age (years)	57.3 (34–88)
Men	56 (62.9%)
Weight (kg)	78.68 (55–107)
Height (cm)	168.14 (150–184)
BMI (Kg/m^2^)	27.87(19.7–34.6)
Smoking	20 (22.5%)
ESRD	2 (2.2%)
Diabetes mellitus	25 (28.1%)
Hypertension	49 (55.1%)
Dyslipidemia	35 (39.3%)
Family history of CAD	24 (27.0%)

Data are expressed as No (%) or mean (Min-Max)

Abbreviations: *BMI* body mass index, *ESRD* end-stage renal disease, *CAD* coronary artery disease

Angiographic findings showed 51 patients (57.3%) with significant coronary lesions in at least one coronary vessel, while 21 patients (23%) had normal coronary arteries with no CAD; details of coronary angiography results are summarized in Table 2.

**Table 2:** Coronary angiography results

LM disease	3 (3.4%)
Significant LAD stenosis	30 (33.7%)
Significant diagonal stenosis	18 (20.2%)
Moderate LAD stenosis	5 (5.6%)
Significant LCX stenosis	20 (22.5%)
Significant OM stenosis	19 (21.3%)
Significant RCA stenosis	31 (34.8%)
Moderate RCA stenosis	9 (10.1%)
Single vessel disease	16 (18.2%)
Two vessel disease	14 (15.7%)
Three vessel disease	21 (23.6%)
Mild CAD	17 (19.1%)
Normal coronary arteries	21 (23.6%)
Significant CAD	51 (57.3%)

Data are expressed as No (%)

Abbreviations: *CAD* coronary artery disease, *LAD* Left anterior descending artery, *LCX* left circumflex artery, *RCA* right coronary artery, *OM* obtuse marginal

Table 3 shows the frequency of coronary risk factors in patients with and without CAD, indicating a significant correlation between these risk factors and significant CAD.

**Table 3:** Frequency of coronary risk factors in patients with and without CAD

Risk factors	Significant CAD	Normal coronary arteries	*P-*value
DM	16 (17.97%)	6 (6.74%)	0.052
HTN	34 (38.2%)	12 (13.48%)	0.002
DLP	24 (26.96%)	10 (11.23%)	0.02
FH	17 (19.1%)	4 (4.49%)	0.007
smoking	15 (16.85%)	5 (5.61%)	0.041

Data are expressed as No (%)

Abbreviations: *CAD* coronary artery disease, *DM* diabetes mellitus, *HTN* hypertension, *DLP* dyslipidemia, *FH* family history of premature CAD

The mean GLS was −16.8% in patients with significant CAD, −16.7 % in SVD patients, −16.8 % in 2VD group, and −17.08% in 3VD population, which was significantly lower compared to the controls group with normal epicardial coronary arteries (*P-*value=0.0001) (Table 4).

**Table 4:** Mean GLS in respect to CAD severity

Involvement of coronary arteries	Mean GLS (%)	***P-***value
Significant CAD	−16.8558±3.78	0.0001
SVD	−16.7±4.07	
2VD	−16.8143±3.39	
3VD	−17.085±3.93	
NECA	−20.4857±2.73	

Abbreviations: *SVD* single vessel disease, *2VD* two-vessel disease, *3VD* three-vessel disease, *NECA* normal epicardial coronary artery

The study findings showed a statistically significant correlation between TLS abnormality and significant lesions in the related coronary artery. Mean TLS of LAD segments was −17.7031 in patients with significant LAD lesions, and it was −20.6676 in patients without significant LAD stenosis (*P-*value=0.005). This correlation was also observed in LCX (*P-*value=0.016) and RCA territory (*P-*value=0.001) (Table 5).

**Table 5:** Correlation of TLS and significant CAD in each coronary artery territory

Coronary artery territories	Number	Mean TLS (%)	***P-***value
LAD segments in patients			0.005
With significant LAD lesion	37	−17.7031	
Without significant LAD lesion	52	−20.6676	
LCX segments in patients			0.016
With significant LCX lesion	30	−15.0267	
Without significant LCX lesion	58	−17.35	
RCA segments in patients			0.001
With significant RCA lesion	31	−15.5548	
Without significant RCA lesion	57	−18.29	

Abbreviations: *CAD* coronary artery disease, *LAD* left anterior descending artery, *LCX* left circumflex artery, *RCA* right coronary artery, *TLS* territorial longitudinal strain

**Fig. 5:**
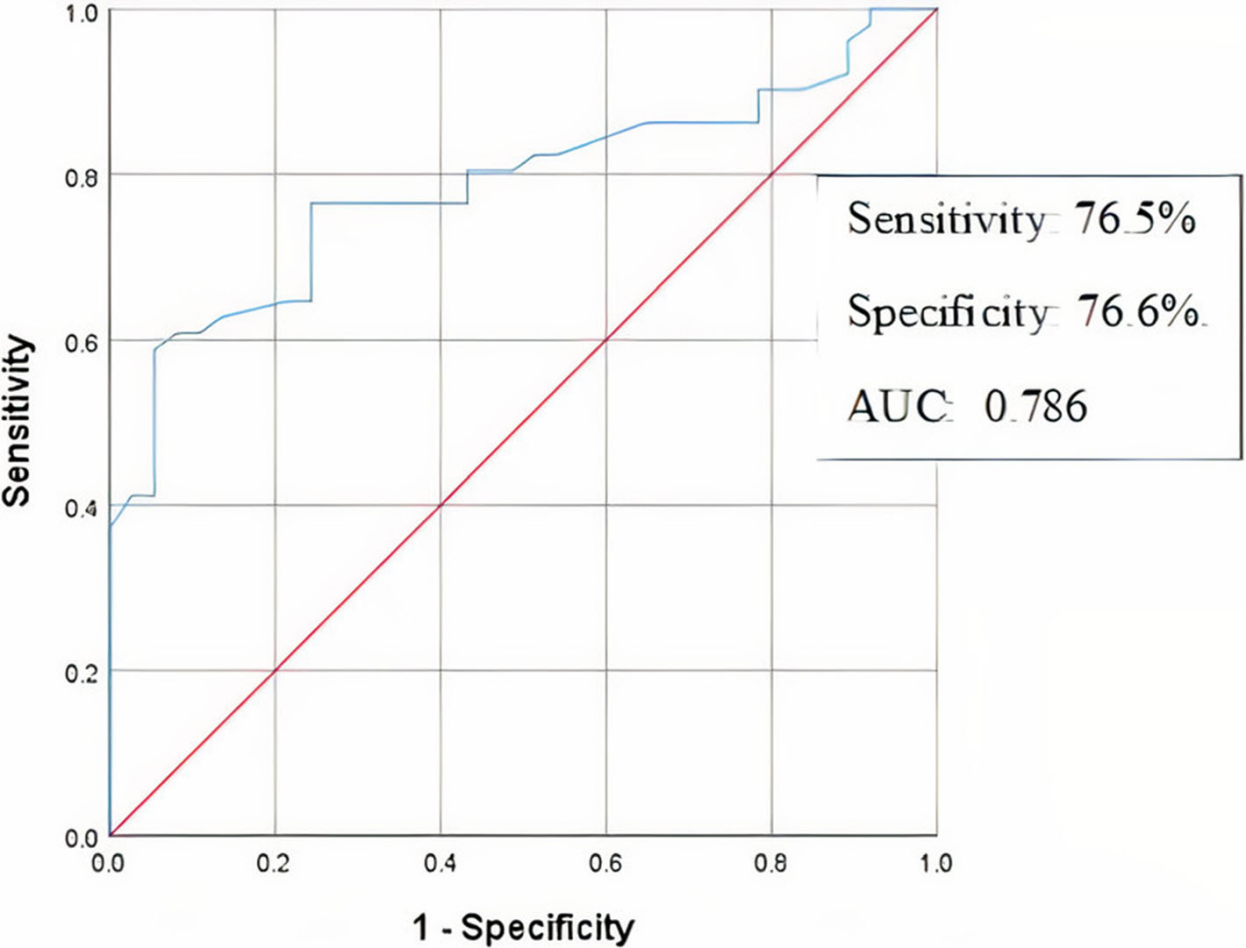
Receiver operating characteristic (ROC) curve analysis (global longitudinal strain ≥ −19.25 may be useful to detect coronary artery disease)

The receiver operating curve (ROC) was used to find the best cut-off point for sensitivity and specificity of GLS in the patients with significant CAD, which was reported as −19.25 with a sensitivity of 76.5% and specificity of 76.6%. The area under the receiver operating curve [(AUC)] was 0.786 (Fig. 5).

### Reproducibility of evidence

For 10% of the study population randomly selected, the inter- and intra-observer variability were calculated, and the results fell in the acceptable range. The inter-observer and intra-observer variability correlation coefficients for GLS and SLS were as follows: Interobserver variability = 0.008 ± 0.301 (mean ± Standard error of mean), Interobserver variability = 0.323 ± 0.137 (mean ± Standard error of mean)

## Discussion

Sub-endocardial fibers are the most sensitive fibers to ischemic events [12]. This study was conducted to prove the correlation between systolic shortening of these fibers in the longitudinal direction and CAD. Biering-Sørensen et al. found that GLS can increase the diagnostic power of exercise tests. Furthermore, they represented a cut-off value for GLS of −18.4% with 74% sensitivity and 89% specificity for detecting significant CAD [12]. Moreover, Radwan and Hussein compared GLS in patients with percent diameter of 70% or greater versus others with less than 70% coronary stenosis, which resulted in statistically significant differences between two groups. Additionally, the cutoff point of −15.6% for GLS showed the highest sensitivity and specificity of 93.1% and 81.8%, respectively [17]. Tsai et al. evaluated GLS, peak segmental longitudinal strain difference, and ratio to peak systolic GLS, which showed lower values in patients with CAD than those with standard coronary angiography. They also showed that using GLS value >−19% as a diagnostic tool for CAD with a 74.7% sensitivity and 80.5% specificity compatible with the present study’s ROC curve results [7]. In confirmation of the previous researches, the present study underscores the significantly lower GLS among patients with significant CAD. Additionally, the ROC curve analysis illustrated a cutoff point of −19.25% with a 76.5% sensitivity and 76.7% specificity, which was somewhere between the figures obtained in the three noted studies.

Recently, a few studies sought to evaluate whether there is a relationship between TLS and coronary stenosis. Casper et al. found that TLS may be of great help to localize significant coronary artery stenosis. They reported a threshold of −19.4%, −16.4%, and −18% for the TLS in LAD, LCX, and RCA segments, respectively, to predict stenosis in related coronary arteries [18]. On the other hand, Zou et al. demonstrated GLS as a good predictor for diagnosing LM or three-vessel CAD in detecting stenotic coronary arteries [19]. These discrepancies encouraged us to run our study to evaluate further the relationship between coronary stenosis and TLS in given territories. Further analysis showed a statistically significant correlation between low TLS and significant CAD in the LAD, RCA, and LCX territories. These results indicate that strain analysis can be used for screening of CAD in patients with no regional wall motion abnormalities at rest and may also help surmise which coronary artery may have been affected.

As expected, this document supports more frequent conventional coronary risk factors (diabetes mellitus, hypertension, hyperlipidemia, smoking, and premature family history of coronary disease) in CAD patients compared to ones with normal epicardial coronary arteries. On the other hand, the mean age of 57.3 years old in the population study could imply the higher prevalence of young CAD in our society arising from ethnic differences.

## Limitations

Our study population was small, and this study considered only the anatomical severity of coronary stenosis in coronary angiography, but not the functional severity of stenosis or coronary microvascular disease, so incorporating the functional significance of coronary stenosis into anatomical characteristics using FFR is recommended for future studies. Furthermore, the obtained GLS cut-off value for identifying LV ischemia should be validated in studies with a large number of CAD patients.

## Conclusion

This study shows that patients with CAD have lower GLS than others with normal coronary arteries. Additionally, subjects with significant stenosis in a specific coronary artery have less harmful longitudinal strain in the coronary artery territory than others with a patent index coronary artery. These speckle-tracking findings may benefit not only the diagnosis of CAD but also in defining the affected coronary arteries, suggesting GLS and TLS as a presumptive non-invasive tool for coronary stenosis detection.

## Data Availability

The datasets generated and/or analyzed during the current study are available in the [Pubmed, Web of Science, Scopus, EM Base] repository.

## Abbreviations

CAD: Coronary artery disease
2DSTE: 2D-speckle-tracking echocardiography
GLS: Global longitudinal strain
TLS: Territorial longitudinal strain
DTI: Doppler tissue imaging
PCI: Percutaneous coronary intervention
ACS: Acute coronary syndrome
SLS: Segmental longitudinal strain
LAD: Left anterior descending
RCA: Right coronary artery
LM: Left main
BMI: Body mass index
ESRD: End-stage renal disease
LCX: Left circumflex artery
DM: Diabetes mellitus
HTN: Hypertension
DLP: Dyslipidemia
FH: Family history of premature CAD
SVD: Single vessel disease
2VD: Two-vessel disease
3VD: Three-vessel disease
NECA: Normal epicardial coronary artery
ROC: Receiver operating curve
